# Partial Protective Effect of Intranasal Immunization with Recombinant *Toxoplasma gondii* Rhoptry Protein 17 against Toxoplasmosis in Mice

**DOI:** 10.1371/journal.pone.0108377

**Published:** 2014-09-25

**Authors:** Hai-Long Wang, Tie-E Zhang, Li-Tian Yin, Min Pang, Li Guan, Hong-Li Liu, Jian-Hong Zhang, Xiao-Li Meng, Ji-Zhong Bai, Guo-Ping Zheng, Guo-Rong Yin

**Affiliations:** 1 Research Institute of Medical Parasitology, Shanxi Medical University, Taiyuan, Shanxi, PR China; 2 Department of Physiology, Key Laboratory of Cellular Physiology Co-constructed by Province and Ministry of Education, Shanxi Medical University, Taiyuan, Shanxi, PR China; 3 Department of Respiratory, the First Affiliated Hospital, Shanxi Medical University, Taiyuan, Shanxi, PR China; 4 Department of Physiology, Faculty of Medical and Health Sciences, University of Auckland, Auckland, New Zealand; 5 Department of Biochemistry and Molecular Biology, Shanxi Medical University, Taiyuan, Shanxi, PR China; Federal University of São Paulo, Brazil

## Abstract

*Toxoplasma gondii* (*T. gondii*) is an obligate intracellular protozoan parasite that infects a variety of mammals, including humans. An effective vaccine for this parasite is therefore needed. In this study, RH strain *T. gondii* rhoptry protein 17 was expressed in bacteria as a fusion with glutathione S-transferase (GST) and the recombinant proteins (rTgROP17) were purified via GST-affinity chromatography. BALB/c mice were nasally immunised with rTgROP17, and induction of immune responses and protection against chronic and lethal *T. gondii* infections were investigated. The results revealed that mice immunised with rTgROP17 produced high levels of specific anti-rTgROP17 IgGs and a mixed IgG1/IgG2a response of IgG2a predominance. The systemic immune response was associated with increased production of Th1 (IFN-γand IL-2) and Th2 (IL-4) cytokines, and enhanced lymphoproliferation (stimulation index, SI) in the mice immunised with rTgROP17. Strong mucosal immune responses with increased secretion of TgROP17-specific secretory IgA (SIgA) in nasal, vaginal and intestinal washes were also observed in these mice. The vaccinated mice displayed apparent protection against chronic RH strain infection as evidenced by their lower liver and brain parasite burdens (59.17% and 49.08%, respectively) than those of the controls. The vaccinated mice also exhibited significant protection against lethal infection of the virulent RH strain (survival increased by 50%) compared to the controls. Our data demonstrate that rTgROP17 can trigger strong systemic and mucosal immune responses against *T. gondii* and that ROP17 is a promising candidate vaccine for toxoplasmosis.

## Introduction

*Toxoplasma gondii* is an obligate intracellular parasite of the phylum *Apicomplexa* [1] that is found worldwide. *T. gondii* has a gigantic intermediate host range that comprises nearly any bird and mammal, including humans. Symptoms associated with *Toxoplasma* infection in humans range from none to severe and can be fatal. For example, *T. gondii* infection is usually asymptomatic but occasionally triggers mild symptoms when *T. gondii* infects immunocompetent hosts. When *T. gondii* infects immunocompromised hosts, such as AIDS patients or malignancy patients, it can lead to severe or even lethal damage [2], [3]. In addition, *T. gondii* infection of livestocks can also result in serious economic losses due to abortion, stillbirth, and neonatal death. Moreover, infected livestock are a major route of *T. gondii* transmission to humans [4]. Therefore, the development of effective and safe methods, such as vaccines [5], to control *T. gondii* infection is crucial for human health and animal husbandry.

Currently, candidate vaccines that have been testing in mice are in the focus of protective antigen selections from membrane-associated surface antigens, excreted-secreted dense granule proteins, rhoptry proteins and micronemal proteins [6]–[8]. Of interests, rhoptry proteins (ROPs) excreted by rhoptries of the apical secretory organelles are involved in parasitic invasion [9]. Some of the ROPs, such as ROP16 and ROP18, are serine-threonine kinases named as ROP kinases (ROPK) and play the role of virulence factors [10]–[13]. Recently, ROP16 and ROP18 have been used as immunogens to vaccinate mice, and enabled the mice to produce considerable cellular and humoral immune responses that partly protected the mice against *T. gondii* infection [14]–[17].

Rhoptry protein 17 (ROP17), which belongs to the ROP2 superfamily, is predicted to be a ROPK [18] and possesses a key ATP-binding domain and conserved residues in its catalytic triad region (KDD) [18], [19] as ROP16 and ROP18 [11], [20]. Our previous study showed that ROP17 has kinase activity because it can phosphorylate c-Jun in HEK 293T cells [21]. Given that ROP16 and ROP18 can induce mice to produce protective immune responses against *Toxoplasma*, we hypothesize that ROP17 is a candidate vaccine for *T. gondii*.

Recombinant *T. gondii* ROP17 (rTgROP17) protein was produced in *Escherichia coli* (*E. coli*) and shown to exhibit specific antigenicity in our previous study [22]. The objective of the present study was to examine the systemic and mucosal immune responses elicited in BALB/c mice after nasal immunization with rTgROP17 and to evaluate the protective efficacy of rTgROP17 against both chronic and lethal challenges with *T. gondii* in mice.

## Materials and Methods

### Mice, parasites and ethics statement

Female BALB/c mice at the age of 6 weeks were purchased from the Institute of Laboratory Animals of the Chinese Academy of Medical Science (Beijing, China) and used for immunization. All of the mice were maintained under standard, pathogen-free conditions. The tachyzoites of the virulent *T. gondii* RH strain were used as a challenge for the immunized mice, and preparations of *T. gondii* genomic DNA were kindly provided by the Health Science Centre of Peking University (Beijing, China). The parasites were maintained and collected from the peritoneal cavity of infected BALB/c mice in our laboratory according a previously described method [23]. All experimental animal procedures were approved by the Laboratory Animal Use and Care Committee of Shanxi Medical University (permit Number: SXMU-2011-16) and the Ethics Committee of Animal Experiments of Shanxi Medical University (permit Number: 20110320-1). All surgeries were performed under sodium pentobarbital anaesthesia, and all possible efforts were made to minimize the suffering of the experimental mice.

### Expression and purification of rTgROP17

Recombinant *T. gondii* ROP17 protein (rTgROP17) was expressed in *E. coli* Rosetta (DE3) strain [22]. Briefly, the open reading frame of TgROP17 gene (GenBank Accession No. AM075203.1) was amplified with a pair of specific primers, and the RT-PCR product was cloned into the prokaryotic expression pGEX-6P-1 vector (Merck Biosciences, Germany). The recombinant pGEX-6P-1/TgROP17 plasmid was transferred into *E. coli* DH5a, and positive clones were selected via double restriction enzyme digestion and DNA sequencing. The successful pGEX-6P-1/TgROP17 construct was transformed into *E. coli* Rosetta (DE3), and its expression was induced with IPTG and analysed with SDS-PAGE. The antigenicity of the resultant rTgROP17 was analysed with rabbit antiserum for *T. gondii* using Western blotting assays.

All of the subsequent purification steps were performed at 4°C. The clear supernatants were applied onto a self-packaged glutathione S-transferase (GST)-affinity column (2 ml Glutathione Sepharose 4B; Qiagen, Germany), and contaminant proteins were removed with cold phosphate buffer saline (PBS). The fusion protein was then eluted with an elution buffer (Tris-HCl, 25 mM; NaCl, 200 mM; DTT, 2 mM; glutathione, 20 mM, pH 7.5). The eluent was concentrated using an Ultrafree 10,000 molecular weight cut-off filter unit (Millipore, USA). The purified protein was analysed by using SDS-PAGE and Coomassie blue R-250 staining.

The endotoxin contained in the rTgROP17 was removed using a ToxinEraser Endotoxin Removal Kit, and the endotoxin level was measured with a Chromogenic End-point Endotoxin Assay Kit (Chinese Horseshoe Crab Reagent Manufactory, Xiamen, China). Less than 0.1 EU/ml of endotoxin was detected in the final protein preparations. rTgROP17 was dialysed against PBS, quantified with the BCA method, filtered through a 0.2 mm-pore membrane and stored at −70°C.

### Intranasal immunization and sample collection

For the immunization experiments, forty female BALB/c mice were randomly divided into 5 groups (8 mice per group). The mice were immunized nasally with 20 µl PBS containing 15, 25, 35 or 45 µg of rTgROP17, which was instilled slowly into both nostrils (10 µl per nostril) with a micropipette. The control mice were given PBS solution only. All animals were vaccinated three times on days 0, 14, and 21 [24].

The samples, including blood and mucosal washes, were collected two weeks after the last immunization (on day 35). The mice were deprived of food for 8 h to deplete the contents of the intestines and were then anesthetised with sodium pentobarbital (1.5%, 0.1 ml/20 g weight, intraperitoneal injection). The blood, nasal washes, vaginal washes and intestinal washes were collected according to published protocols [25]. All samples were stored at −20°C until assayed for antibody titres and cytokines levels.

### Specific IgG and IgA detection

Serum samples and mucosal washes were tested for the presence of specific IgG and secretory immunoglobulin A (SIgA) using enzyme linked immunosorbent assays (ELISAs). Ninety-six well polystyrene plates (Corning) were coated with 7.5 µg/ml rTgROP17 (100 µl/well) in PBS overnight at 4°C. The plates were washed with PBS containing 0.05% Tween20 (PBST) and blocked for 1 hour at 37°C in PBS containing 5% BSA and then washed with PBS. Serum samples were diluted 1∶200 in PBS, mucosal washes without dilutions were incubated at 4°C overnight in different wells of the coated 96 well plates at 100 µl/well. Next day, the wells were washed and incubated with 100 µl of HRP-labelled goat anti-mouse antibody (AbD Serotec; diluted 1∶2500 in PBS) for serum specific IgG for 1 h at 37°C. The wells were washed extensively, incubated with orthophenylene diamine (Sigma, USA) and 0.15% H_2_O_2_ for 30 min, and enzyme reactions were terminated by the addition of 50 µl of 1 M H_2_SO_4_. Optical density (OD) was measured in an ELISA plate reader (Epoch Multi-Volume Spectrophotometer System, Biotek, USA) at 492 nm. All samples were analysed in triplicate for at least three independent experiments.

### Cytokines assays

Spleen cells were obtained as described previously [24], [25] and cultured in flat-bottom 24-well microtitre plates at 1.5×10^6^ cells/well. Following 24 or 96 h of stimulation with rTgROP17 at 10 µg/ml, tissue culture media were collected and assayed for interleukin-2 (IL-2), IL-4 and IL-5 at 24 h [26] and for interferon-gamma (IFN-γ) at 96 h. The concentrations of IL-2, IL-4, IL-5 and IFN-γ were determined with a commercial ELISA kit (PeproTech, USA) according to the manufacturer’s instructions. All assays were performed in triplicates. The sensitivity limits of detection for IL-2, IL-4, IL-5 and IFN-γ were 16, 16, 32 and 23 pg/ml, respectively.

### Lymphocyte proliferation assay

Splenocyte prolifereations upon the stimulation of rTgROP17 and controls were determined with the colorimetric Cell Counting Kit-8 (CCK-8, Dojindo Laboratories; Kumamoto, Japan) according to the manufacture’s instruction. Isolated spleen cells were plated in flat-bottom 96-well microtitre plate at a density of 5×10^5^ cells/well, and cultured in the presence of rTgROP17 (10 µg/ml), Concanavalin A (Con A; 5 µg/ml; Sigma; positive control) or PRIM 1640 medium alone (negative control) at 37°C in a 5% CO_2_ incubator. After 72 h, 10 µl of CCK-8 reagent was added to each well, the cells were cultured for further 4 h, and absorbance at 450 nm was determined to evaluate cell proliferation. The stimulation index (SI) was calculated as the ratio of the average OD_450_ value of the wells containing antigen-stimulated cells to the average OD_450_ value of the wells containing cells with medium only. All assays were performed in triplicates for at least three independent experiments.

### Challenge procedure

Challenge procedure was performed in two sets of independent experiments. In each set of experiments, forty female BALB/c mice at 6 weeks of age were randomly divided into two groups (20 mice per group) and vaccinated intranasally with 35 µg of rTgROP17 or GST control (Sigma, USA) in 20 µl volumes on days 0, 14, and 21 as described above. On day 14 after the final immunization, 8 mice from each group were orally challenged via a feeding needle with 1×10^4^ tachyzoites of the RH strain for chronic assay, and 12 mice from each group were similarly challenged with 4×10^4^ tachyzoites for acute infection. One month later, the chronically infected mice were anaesthetized with sodium pentobarbital, and the numbers of tachyzoites in the murine livers and brains were measured using real-time PCR assays. For the acute challenge infection, the times until death of the challenged mice were monitored and recorded for 30 days following the parasite challenge to compute the survival rates. During this period, the infected mice were closely monitored and recorded at 8 am, 2 pm and 8 pm daily for their physical appearance such as rough coat, decrease in appetite, weakness/inability to obtain feed or water, depression. When any of these syndromes happened, the mice would be moved to an isolated cage for further husbandry. If obvious sufferings such as struggling or whining were observed, the mice would be anesthetized by inhaling ether and euthanized.

### DNA extraction and real-time PCR assay

To evaluate the tachyzoite loads, genomic DNA from the liver and brain samples (100 mg each) of the chronically infected mice and purified parasites were extracted using a UniversalGen DNA Kit (CWBIO, China) according to the manufacturer’s instructions. The forward and reverse primer sequences for the surface antigen 1 (SAG1) gene of the tachyzoite were 5′-CTGATGTCGTTCTTGCGATGTGGC-3′ and 5′-GTGAAGTGGTTCTCCGTCGGT GT-3′, respectively. PCR was performed using the Applied Biosystems Real-Time PCR Instrument and SYBR green fluorescence detection. Each reaction mixture contained 10 µl of UltraSYBR Mixture (CWBIO, China), 0.4 µl of each primer (20 µM), 1 µl of DNA template and 8.2 µl of sterile distilled water. Sterile water was used as a negative control, and the DNA extracted from 500 tachyzoites of the *T. gondii* RH strain was used as a positive control. All reactions were performed in triplicates and incubated at 95°C for 1 min, followed by 40 cycles of incubation at 95°C for 5 s, 60°C for 15 s and 72°C for 10 s [27].

The qPCR threshold cycle (Ct) value obtained with DNA samples from a range of serial 10-fold dilutions (5×10^0^–5×10^7^/ml) of RH strain tachyzoites was used to calculate the numbers of parasites in the brain and liver samples. The tachyzoite loads were presented as the estimated mean values of the quantities of tachyzoites per gram of tissue.

### Statistical analysis

Data were expressed as mean ± standard error for three or more independent experiments. Statistical comparisons between the test and control groups were performed with one-way analyses of variance (ANOVA) using the SPSS13.0 Data Editor software (SPSS Inc., Chicago, IL). *P* values<0.05 were taken as statistically significant.

## Results

### Purification of rTgROP17 with GST-affinity chromatography

**Figure 1:**
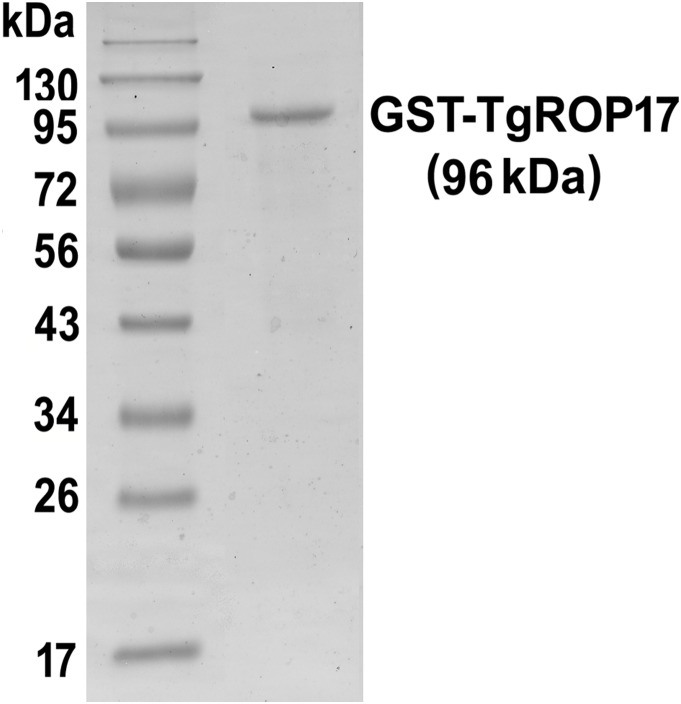
SDS-PAGE analysis of the rTgROP17 protein. Purified rTgROP17 protein was analysed by 12% SDS-PAGE and Coomassie blue staining. The purity of the rTgROP17 was greater than 95%.

Expression of rTgROP17 was successfully achieved in *E. coli* [22]. To obtain the purified recombinant protein, we performed a GST-affinity chromatography. The isolated protein with a GST Tag at its N-terminal was approximately 96 kDa, water-soluble, and 95% pure as determined by SDS-PAGE gel analysis (Fig. 1).

### rTgROP17 vaccination induces systemic immune responses

To evaluate the systemic immune responses in rTgROP17**-** immunized mice, the levels of antigen-specific IgG, IgG1 and IgG2a antibodies in the sera and in the spleen cell supernatants were detected using ELISA. Splenic lymphocyte proliferation was determined with the CCK-8 method on day 14 after the final vaccination.

**Figure 2:**
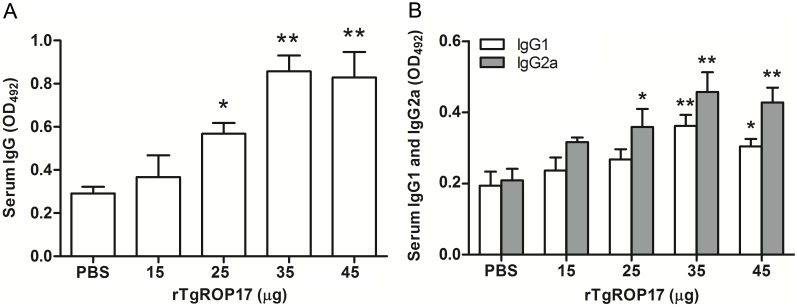
Determination of specific anti-rTgROP17 antibody responses in serum samples from vaccinated and control mice by ELISA. The levels of both specific total IgG (A) and IgG isotype (B) antibodies in the sera of the BALB/c mice were determined by ELISA with rTgROP17 as the bound target two weeks after the final immunization. The results are expressed as the mean value ± SD of OD_492_ (n = 8) from three independent experiments. *P<0.05, **P<0.01 relative to PBS control groups.

As shown in Fig. 2A, the total IgG antibody productions of the mice immunized with 35 and 45 µg rTgROP17 were significantly higher than those of the control group (*P*<0.01) but not significantly different from each other (*P*>0.05). The results also revealed that 25 µg rTgROP17 but not 15 µg rTgROP17 elicited elevated IgG antibody levels compared to the control groups (*P*<0.05). Moreover, both IgG1 and IgG2a were detected in the sera of all the mice immunized with rTgROP17, particularly in those immunized with 35 and 45 µg rTgROP17 (Fig. 2B). While 25 µg TgROP17 induced significantly higher levels of IgG2a but not IgG1 than those of the control groups (*P*<0.05). No difference of IgG1 or IgG2a responses was found between 15 µg TgROP17 and the control groups (*P*>0.05). In general, greater levels of TgROP17-specific IgG2a were detected than those of its IgG1 counterpart. These results indicate that rTgROP17 immunization trigers a Th1-type systemic immune responses in the mice.

We next analysed the cell-mediated immunity produced in the immunized mice by indirectly evaluating the amounts of cytokines (IFN-γ, IL-2, IL-4 and IL-5) released in the culture media of isolated spleen cells from the rTgROP17-immunized mice. Compared to the PBS controls, the spleen cells from the mice vaccinated with 35 and 45 µg rTgROP17 released significantly higher levels of IFN-γ and IL-2 (*P<*0.01), but there was no significant difference between the 35 and 45 µg groups (*P*>0.05) (Table 1). While 25 µg rTgROP17 also stimulated the production of IFN-γ and IL-2 over the PBS control group (*P*<0.05), 15 µg rTgROP17 did not stimulate IFN-γ or IL-2 production (*P*>0.05). Similarly, the levels of IL-4 secreted by the splenocytes of the 35 and 45 µg rTgROP17-immunized mice were also significantly higher than those of the PBS control group (*P*<0.05) but not the 15 and 25 µg groups (Table 1). However, no significant difference was observed for the levels of IL-5 between the rTgROP17-vaccinated and PBS control mice (*P*>0.05). Therefore, these data further suggest that rTgROP17 induces a Th1-type immune responses that is correlated with expressions of IFN-γ and IL-2 but not IL-4 and IL-5.

**Table 1:** Lymphocyte proliferation and cytokine production by splenocytes stimulated with rTgROP17.

Groups^a^	Lymphocyte SI	Cytokine production (pg/ml)^b^			
		IFN-γ	IL-2	IL-4	IL-5
PBS	1.07±0.15	49.63±7.42	80.81±4.09	78.91±8.72	81.48±9.75
15 µg rTgROP17	1.28±0.10	64.84±6.59	96.05±23.88	86.22±13.64	86.37±7.97
25 µg rTgROP17	1.63±0.13*	79.46±12.38*	119.76±12.25*	93.15±10.25	85.29±16.41
35 µg rTgROP17	2.38±0.16**	125.45±14.12**	136.69±16.89**	122.17±13.08*	89.47±10.52
45 µg rTgROP17	2.29±0.19**	117.43±11.29**	133.66±12.26**	118.20±11.78*	83.59±11.26

a *n* = 8 per group.

b Splenocytes were harvested from the mice 2 weeks after the final immunization. The results are presented as the arithmetic means ± the standard errors of three independent experiments. The values of IFN-γ are for 96 h, and the values of IL-2, IL-4 and IL-5 are for 24 h.

*P<0.05 and **P<0.01 compared to the PBS group.

To assess the proliferative immune responses to rTgROP17, splenocytes from the immunized mice were also prepared two weeks after the third immunization. The splenocyte stimulation indices (SIs) of the mice immunized with 35 and 45 µg rTgROP17 were significantly greater than those of the mice immunized with 15 µg rTgROP17 or PBS control (*P<*0.01) (Table 1). While the SI of the 25 µg group was also greater than that of the PBS control (*P<*0.05), no significant difference was found for the SIs of the 15 µg group and PBS controls (*P*> 0.05). Of note, comparable levels of proliferation were observed in response to ConA for all the splenocytes from all experimental and control mice mice (data not shown). These results demonstrate that nasal administration of rTgROP17 is able to trigger systemic cell-mediated immunity in mice.

### rTgROP17 vaccination induces mucosal immune responses

**Figure 3:**
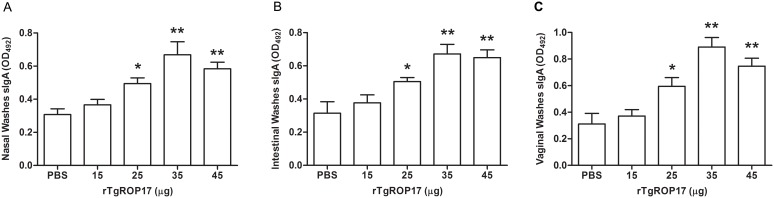
Nasal rTgROP17 immunization of mice induces specific SIgA antibody responses in their mucosal washes. The SIgA antibody titres in the mucosal washes of the rTgROP17-immunised mice were determined by ELISA two week after the final immunization. High levels of SIgA in (A) the nasal washes, (B) intestinal washes and (C) vaginal washes were detected in rTgROP17-immunized mice compared to those of the PBS control mice. The results are expressed as the mean value ± SD of OD_492_ (n = 8) from three independent experiments. *P<0.05, **P<0.01 relative to PBS control groups.

To determine whether the mice intranasally immunized with rTgROP17 developed mucosal immune responses, specific SIgA levels in their mucosal washes were tested with ELISA two weeks after the final immunization. As shown in Fig. 3, higher levels of SIgA were detected in the nasal, vaginal and intestinal washes of rTgROP17-immunized mice compared to those of their PBS controls. In particular, 35 and 45 µg rTgROP17 elicited much higher SIgA antibody titres than those of the controls in all three different mucosal washes (*P<*0.01), and the SIgA antibody titres of the 25 µg group was also higher than those of the PBS controls (*P<*0.05). These data indicate that nasal rTgROP17 immunization in mice can evoke strong mucosal immune responses at their nasal, intestinal and vaginal mucosal sites.

### Protection of mice against oral challenge with *T. gondii* RH strain

**Figure 4:**
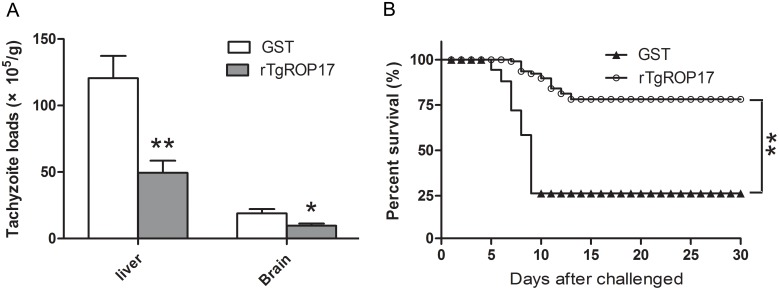
The tachyzoite burdens and survival rates of rTgROP17-immunised mice after oral challenge with *T. gondii*. The mice were nasally immunized with rTgROP17 or GST control. Two weeks after the final immunization, the mice were orally challenged with 1×10^4^ tachyzoites (A) or 4×10^4^ tachyzoites (B) of the *T. gondii* RH strain. (A) The liver and brain tachyzoite burdens of two groups of mice (n = 8 per group) were evaluated one month after the challenge, and (B) the survival rates of other two groups of mice (n = 12 per group) were monitored daily for 30 days following the challenge. The values are presented as mean ± SD from two independent experiments. *P<0.05, **P<0.01 relative to GST control groups.

To evaluate whether rTgROP17 immunization could potentially provide protection against *T. gondii* chronic infection, the numbers of liver and brain tachyzoites in the mice were tested one month after peroral challenge (1×10^4^ tachyzoites of RH strain) using a real-time PCR assay. The tachyzoite loads in the brains and livers of the mice immunized with 35 µg rTgROP17 were significantly reduced to 59.17% (*P*<0.01) and 49.08% (*P*<0.05) of the loads found in the GST-treated control mice, respectively (Fig. 4A).

To further determine whether rTgROP17 vaccination protected the mice against lethal *T. gondii* RH strain infection, the mice were orally challenged with 4×10^4^ tachyzoites 2 weeks after the final immunization, and their survival rates were recorded daily for 30 days following the challenge. As shown in Fig. 4B, the survival rates of the mice immunized with 35 µg rTgROP17 were significantly increased (75%) on the 30th day after the challenge when compared to those of the GST-treated control mice (25%) (*P*<0.01). Survival of rTgROP17-immunized mice was three times over the GST-treated mice. The death time of the control mice ranged from 5 to 9 days, while the mice immunized with rTgROP17 died between days 10 and 14 after the challenge. These results suggest that rTgROP17 vaccination can protect the mice against *T. gondii* infection through reductions in their tissue loads of the tachyzoites and increases of their survival rate and time.

## Discussion

*Toxoplasma* is an obligate intracellular parasite that requires active invasion of a host cell for replication [28]. The invasion process is punctuated by waves of parasite secretion [2], [3]. During the invasive stages of *Toxoplasma* infection, there are three sets of secretory organelles, i.e., micronemes, rhoptries and dense granules, which release proteins that are essential to intracellular survival [29]. Rhoptry proteins (ROPs) are introduced into a host cell during the invasion and are involved in the determination of the virulence of parasites [30]. Thus far, most ROPs have been found to show clear homology with protein kinases [18], [31], [32]. Some ROPs, such as ROP16 and ROP18, are active kinases, while others are pseudokinases. ROP16 is a tyrosine kinase that phosphorylates the key tyrosine for the activation of STATs to regulate many immune response genes [11], [33]–[35]. ROP18 is a serine/threonine kinase that can phosphorylate the immunity-related GTPases (IRGs) and maintain the integrity of the PVM to protect the inside parasites from being destroyed [20], [36]. Due to the key biological role of rhoptries, some ROPs, including ROP16 and ROP18, have recently become candidate vaccines for the prevention of toxoplasmosis [37]. Moreover, ROP16- and ROP18-immunised mice can induce cellular and humoral immune responses that protect the mice against *T. gondii* infection [14]–[17].

ROP17 is predicted to be a serine/threonine kinase because it possesses the catalytic aspartate and the glycine loop responsible for the stabilisation of the αβ-phosphate of adenosine triphosphate (ATP) [11], [18], [19]. Our previous study showed that ROP17 can phosphorylate c-Jun in HEK 293T cells and thus demonstrated its kinase activity [21] In the present study, we demonstrated that nasal delivery of the purified recombinant *Toxoplasma gondii* ROP17 (rTgROP17) protein to BABL/c mice effectively reduced tachyzoite loads in their brain and liver tissues, prolonged their survival time and increased their survival rate following oral challenges with the virulent RH strain of *T. gondii* in association with enhanced systemic and mucosal immune responses and also cellular-mediated immune response of the rTgROP17-immunised mice.

*Toxoplasma* infects humans and animals primarily through the mucosal surfaces of the digestive tracts. Additionally, *Toxoplasma* can invade all nucleic cells and disseminate throughout the hosts. Therefore, the development of a potential vaccine against *Toxoplasma* infection requires the induction of protective responses at mucosal surfaces and also the induction of systemic protective immune responses in the systemic compartment. Recently, vaccination at local mucosal surfaces, particularly the nasal surface, has been proven to be a promising and rational approach for vaccine development [25], [38]. A number of lines of evidence also indicate that specific SIgA plays a protective role against many pathogens which colonise mucosal tissues or invade the host organism by crossing mucous membranes [39]–[41]. Indeed, SIgA serves as the first line of defence in protecting the intestinal epithelium from enteric toxins and pathogenic microorganisms like *Toxoplasma* [42], [43]. Our results showed that the antigen-specific SIgA antibody responses in the nasal, vaginal and intestinal washes were signifantly increased in the mice which were immunized nasally with 35 µg rTgROP17. These findings demonstrated that nasal immunization of mice with rTgROP17 evokes strong SIgA immune responses in mucosal sites for the initiation or generation of protective immunity against *Toxoplasma*.

One of the goals of a vaccination procedure is to be able to appropriately direct the T helper response. Naturally, the Th1 immune response is predominant when *T. gondii* infects a host [44]. Immunisation with rTgROP17 induced a mixed Th1/Th2 response with a slightly greater increase in the anti-*T. gondii* antibodies of the Ig2a isotype (Th1) than the IgG1 (Th2) isotype (Fig. 2B). Spleen cells from rTgROP17-immunized mice exhibited enhanced proliferation potential and secreted more IFN-γ, IL-2 and IL-4 but not IL-5 than those of the PBS control mice, suggesting that rTgROP17 enhanced Th1 and Th2 cell-mediated immunity with a Th1 type predominance (Table 1). These findings demonstrate that intranasal immunization with rTgROP17 can potentiate humoral and cellular Th1 immune responses in BALB/c mice. As is well known, the modulation of the Th1-type response plays a major deterministic role in the induction of cell-mediated immune responses and the control of acute *T. gondii* infections [45].

There is evidence that recombinant protein vaccine can induce stronger immune responses and better survival protections against acute *toxoplasmosis* than their DNA vaccination counterparts [46]. In recent years, tremendous efforts have been made toward identifying and verifying recombinant protein vaccine candidates against *T. gondii* infection, including recombinant rSAG1 [47], [48], rROP2 [49], [50], rROP4 [50], rGRA1 [46], rGRA4 [49], and rGRA6 [51] protein vaccines. These protein vaccines produce partial protective efficacy or extend the survival times of mice challenged with a lethal *T. gondii*. Our previous studies have also shown that intranasal immunization with rPDI and rACT increases the survival rates and prolongs life spans of the experimental mice [24], [25].

Although encouraging progress has been made, there is still little to offer as protection against toxoplasmosis. One of the problems encountered in the field of research in the development of a vaccine against *T. gondii* is the lack of efficacious protective antigen candidates [52]. In the present study, the protective effect of rTgROP17 protein against *T. gondii* infection was evaluated in mice. The vaccinated mice displayed significant protection against lethal infection of the virulent RH strain with a 50% increase of survival rate over the GST controls. Significant reductions of parasite burdens was also observed in the liver (59.17%) and brain (49.08%) tissues of the rTgROP17-immunised mice (Fig. 4). These data indicate that rTgROP17 offers better protection of mice against *T. gondii* infection compared to the ROP16 and ROP18 used as DNA vaccine candidates which only extended the life span but not the survival rate of *T. gondii-*infected mice [10], [14]–[17].

The antigen delivery pathway is a key parameter for the induction of protective immune responses by vaccines against intracellular pathogens [53]. Most candidate vaccines have been given parenterally to induce systemic immunity, which can control toxoplasmosis efficiently [54], [55]. Several studies have shown that intranasal vaccination is an effective regimen for the stimulation of mucosal immune responses in the mucosal effective sites, including the gut, genital tract, and nose cavity [56], [57]. In the present study, intranasal immunization with rTgROP17 not only induced strong mucosal immune responses in the nasal, vaginal and intestinal mucosal sites, but also induced cell-mediated immunity that included considerable levels of IFN-γand IL-2. To further enhance Th1 cell-mediated immunity and IFNs, different vaccine adjuvants, such as cholera toxin (CT) and IL-2 [23], will be considered in further studies.

In addition, it has yet to be determined whether the immune response induced by ROP17 of the type I *T. gondii* could be effective against type II or type *T. gondii* strains. We compared the amino acid sequences of ROP17s from the RH strain (type I), the ME 49 strain (type II) and the VEG strain (type). Over 99% sequence identity of the ROP17s was found across the three genotypes of *T. gondii* (Figure S1), suggesting that their genetic variation is very low and that similar ROP17 proteins are expressed by the type I, type II and type strains; thus, these proteins may stimulate similar immune responses. This issue will be experimentally investigated in further studies.

On the other hand, the recombinant rTgROP17 protein we used in this study was GST- tagged, and therefore GST was used as a control in the immunization and *T. gondii* challenge assays. Although our data demonstrated that the mice immunized with GST alone exhibited higher tachyzoite burdens and lower survival values compared with those immunized with GST-rTgROP17, antibodies directed against the GST portion of the GST-fusion protein have also occasionally be detected [58]. The use of recombinant ROP17 without the GST fusion partner or as a fusion to another small carriers, such as the His-binding protein [24] would - increase the specificity of the vaccine and reduce the number of animals used in similar future studies.

In summary, our study suggests that intranasal immunization of mice with rTgROP17 can induce both systemic and local immune responses to provide protection against lethal *T. gondii* infections through reduction of the tachyzoite burdens in the host tissues and increases of the animal survivals. We conclude that ROP17 is a promising vaccine candidate against infection with *T. gondii*.

## Supporting Information

Figure S1 **Alignment of TgROP17 amino acid sequences in three strains of** ***T. gondii*** **.** The amino acid sequences of ROP17s from three strains of *T. gondii* were obtained from the Internet (http://www.ncbi.nlm.nih.gov). The protein accession numbers are as follows: CAJ27112 for the RH strain, type I; EPT29356 for the ME 49 strain, type II; and ESS32210 for the VEG strain, type III. The alignment was performed using DNAMAN software (Lynnon, Quebec, Canada). Homology levels are indicated by the colours of: black, 100%; and blue, 50%. Over 99% of sequence identity is found for the ROP17s across the three genotypes of *T. gondii*.
(TIF)
