# Supplementary figures and images for: Partial Protective Effect of Intranasal Immunization with Recombinant Toxoplasma gondii Rhoptry Protein 17 against Toxoplasmosis in Mice
Source: PLoS One. 2014 Sep 25;9(9):e108377. doi: 10.1371/journal.pone.0108377 (PMC4177930; doi:10.1371/journal.pone.0108377)

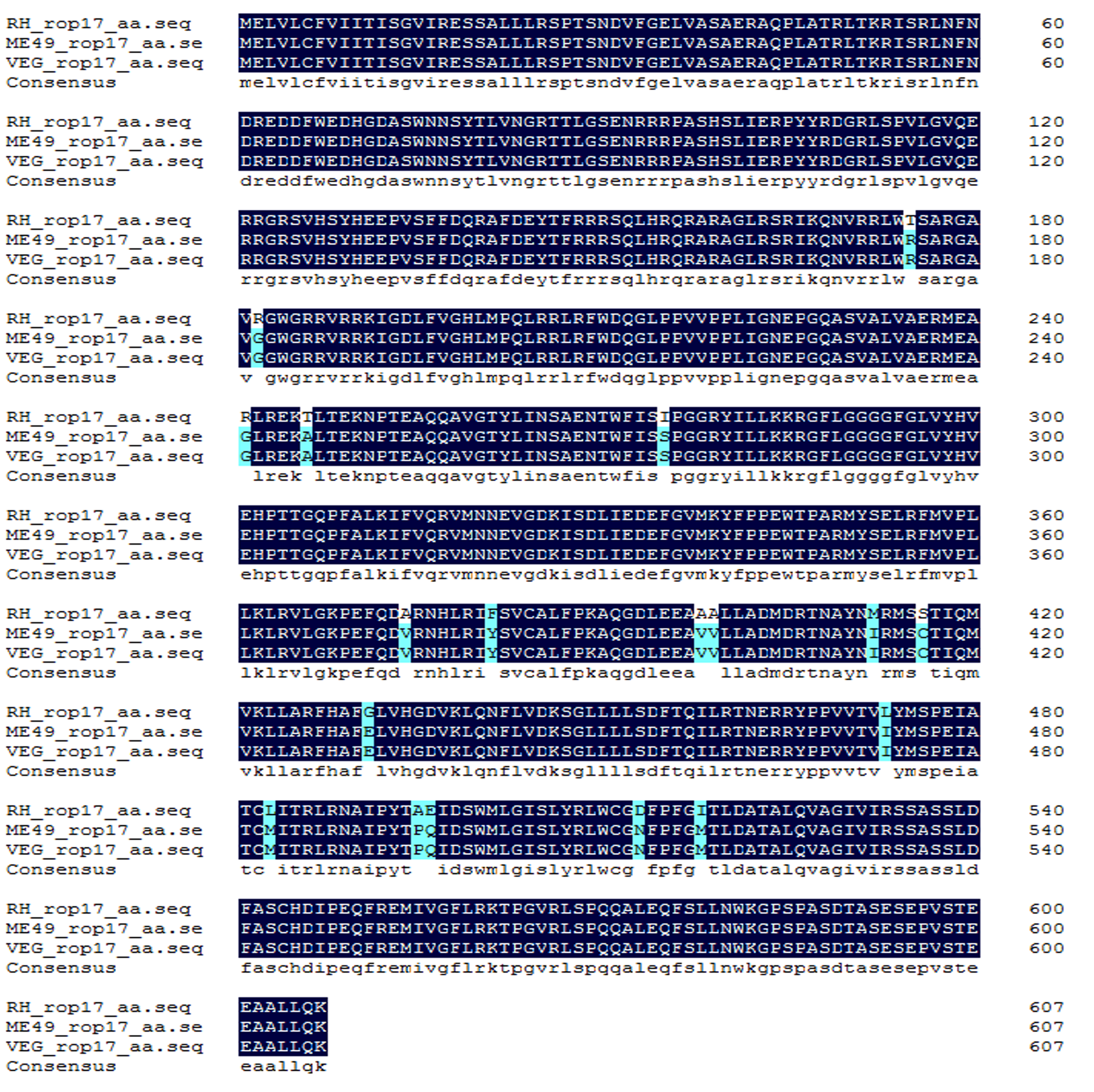

Supplement: Figure S1 — Alignment of TgROP17 amino acid sequences in three strains of T. gondii . The amino acid sequences of ROP17s from three strains of T. gondii were obtained from the Internet (http://www.ncbi.nlm.nih.gov). The protein accession numbers are as follows: CAJ27112 for the RH strain, type I; EPT29356 for the ME 49 strain, type II; and ESS32210 for the VEG strain, type III. The alignment was performed using DNAMAN software (Lynnon, Quebec, Canada). Homology levels are indicated by the colours of: black, 100%; and blue, 50%. Over 99% of sequence identity is found for the ROP17s across the three genotypes of T. gondii. (TIF) [file pone.0108377.s001.tif]
